# Rising temperatures threaten pollinators of fig trees—Keystone resources of tropical forests

**DOI:** 10.1002/ece3.9311

**Published:** 2022-09-17

**Authors:** Lisette van Kolfschoten, Lovisa Dück, Martin I. Lind, K. Charlotte Jandér

**Affiliations:** ^1^ Plant Ecology and Evolution, Department of Ecology and Genetics Evolutionary Biology Centre Uppsala University Uppsala Sweden; ^2^ Smithsonian Tropical Research Institute Ancon Panama; ^3^ Animal Ecology, Department of Ecology and Genetics Evolutionary Biology Centre Uppsala University Uppsala Sweden

**Keywords:** *Ficus*, fig wasp, global warming, life span, mutualism, pollination

## Abstract

Pollinating insects are decreasing worldwide in abundance, biomass, and species richness, affecting the plants that rely on pollinators for fruit production and seed set. Insects are often sensitive to high temperatures. The projected temperature increases may therefore severely affect plants that rely on insect pollinators. Highly specialized mutualisms are expected to be particularly vulnerable to change because they have fewer partner options should one partner become unavailable. In the highly specialized mutualism between fig trees and their pollinating fig wasp, each fig species is pollinated by only one or a few wasp species. Because of their year‐round fruit production, fig trees are considered a keystone resource for tropical forests. However, to produce fruits, wild fig trees need to be pollinated by fig wasps that typically travel a long one‐way trip from the tree donating pollen to the tree receiving pollen. In a few previous studies from China and Australia, increasing temperatures dramatically decreased fig wasp lifespan. Are these grim results generalizable to fig mutualisms globally? Here, we use survival experiments to determine the effect of increasing temperature on the lifespan of Neotropical fig wasps associated with five common Panamanian *Ficus* species. Experimental temperatures were based on the current daytime mean temperature of 26.8°C (2SD: 21.6–31.7°C) and the predicted local temperature increase of 1–4°C by the end of the 21st century. We found that all tested pollinator wasp species had a significantly shorter lifespan in 30, 32, 34, and 36°C compared to the current diurnal mean temperature of 26°C. At 36°C pollinator median lifespan decreased to merely 2–10 h (6%–19% of their median lifespan at 26°C). Unless wasps can adapt, such a dramatic reduction in lifespan is expected to reduce the number of pollinators that successfully disperse to flowering fig trees, and may therefore jeopardize both fruit set and eventually survival of the mutualism.

## Graphical abstract

We determine the effect of the predicted temperature increases on the lifespan of five different Neotropical fig wasps. All tested pollinator wasp species had a significantly shorter lifespan in 30, 32, 34 and 36°C compared to the current diurnal mean temperature of 26°C. Unless wasps can adapt, such a dramatic reduction in lifespan is expected to reduce the number of pollinators that successfully disperse to flowering fig trees, and may therefore jeopardize both fruit set and eventually survival of the mutualism.

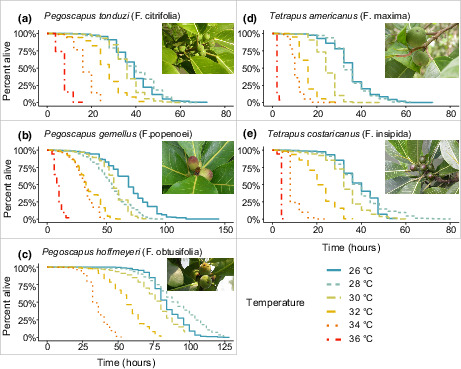

## 1 INTRODUCTION

Worldwide, pollinators are decreasing in occurrence, diversity, and abundance (Hallmann et al., 2017; Potts et al., 2010; Sánchez‐Bayo & Wyckhuys, 2019; Van Swaay et al., 2015; Wagner et al., 2021; Zattara & Aizen, 2021). Causes for this decline include habitat loss, increased pesticide use, and global warming (IPBES, 2016; Kearns et al., 1998; Memmott et al., 2007; Vanbergen & Garratt, 2013). Nearly 90% of flowering plants depend on pollinators for fruit production and seed set (Ollerton et al., 2011), and the number of available pollinators directly influence the seed set of the plant (Ågren, 1996; Robertson et al., 1999). Losing pollinators therefore would have a great effect on plant communities and other organisms dependent on plants (Biesmeijer, 2006; Brosi & Briggs, 2013; Kearns et al., 1998).

Although plants themselves may tolerate rising temperatures, pollinators have quite different physiologies from plants and may be negatively affected by high temperatures (Angilletta & Angilletta, 2009; Suttle et al., 2007). Most pollinators are insects, and studies show that insects can be negatively impacted by high temperatures, having reduced lifespans, reduced fecundity, or increased thermoregulating behavior (Durak et al., 2020; Feder et al., 1997; Mech et al., 2018; Wynants et al., 2021). However, most studies on pollinators measure physiological constrains at maximum viable temperatures (Käfer et al., 2012; Maebe et al., 2021; Maia‐Silva et al., 2021; Oyen & Dillon, 2018; Sánchez‐Echeverría et al., 2019) rather than lifespan at elevated, realistic temperatures (Jevanandam et al., 2013; Nasir et al., 2019). Measuring the actual lifespan of pollinating insects is highly relevant as this can determine their capacity to perform pollination services. Here, we study the effect of temperature on pollinator lifespan, a direct correlate to pollinator dispersal success in the mutualism between fig trees and their pollinating fig wasps.

**FIGURE 1:**
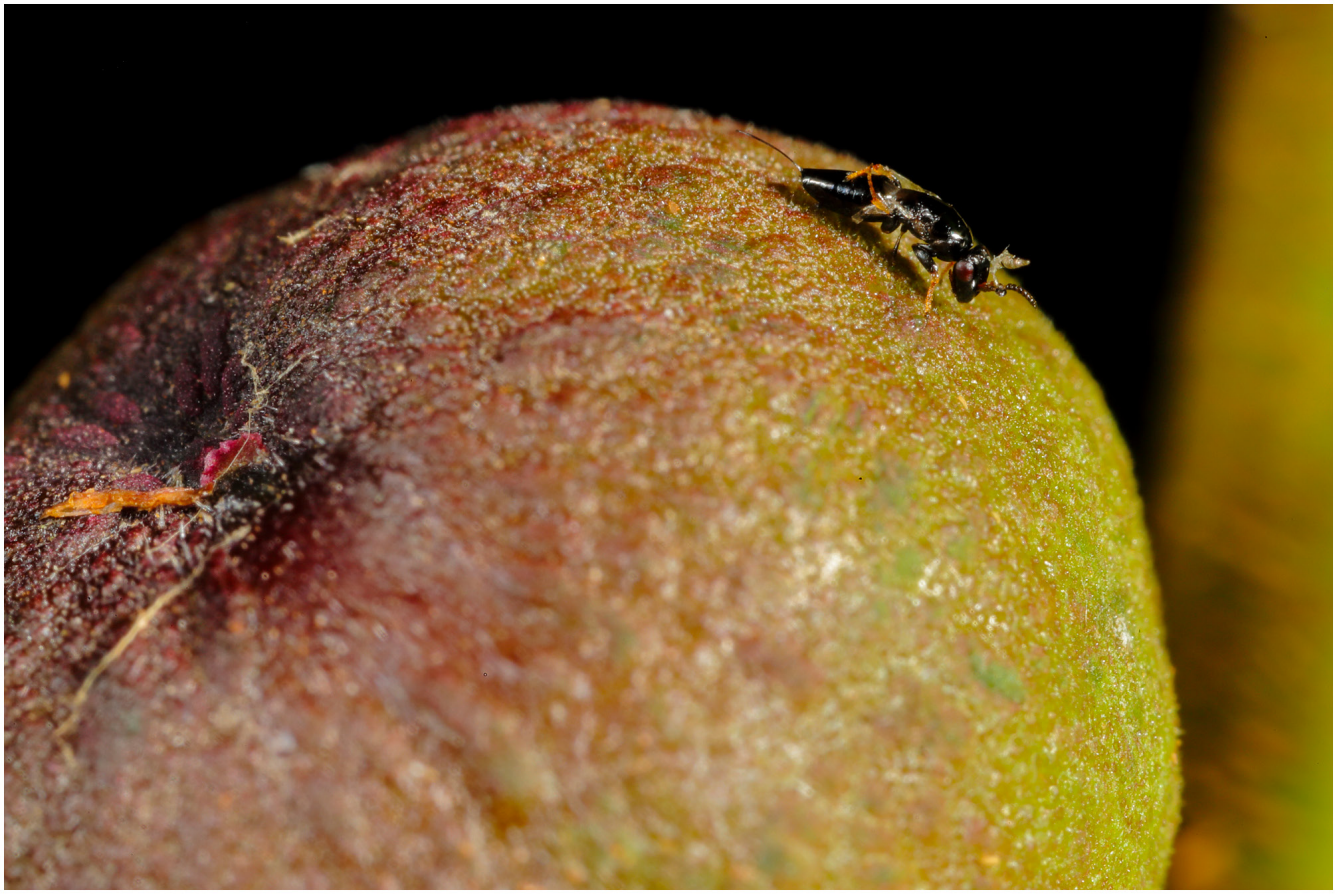
A female fig wasp (*Tetrapus americanus*), pollinator of *Ficus maxima*, has just emerged from her natal fig and is cleaning herself, getting ready for the long one‐way flight to a flowering tree where she can lay her eggs. Photograph by Christian Ziegler (www.christianziegler.photography).

Fig trees are keystone resources in tropical forests globally because by fruiting asynchronously they produce ripe figs year‐round. Up to 70% of rainforest birds and mammals eat figs (Shanahan et al., 2001). To produce fruits, wild fig trees need to be pollinated by fig wasps. Fig trees and fig wasps have been coevolving for 80–90 million years, with currently over 750 species of fig trees globally (Cruaud et al., 2012; Datwyler & Weiblen, 2004). Each species of fig tree can only be pollinated by one or a few species of fig wasps (Figure 1), and fig wasps can only lay their eggs in fig flowers (Herre, 1989; Herre et al., 2008; Wiebes, 1979). Fig wasp larvae develop inside galled fig flowers, and collect pollen from their natal fig before dispersing to a different tree to pollinate and lay their eggs (Herre et al., 2008).

Fig wasps are tiny (1–3 mm), extremely short‐lived (2–3 days on average), and disperse long distances, in central Panama on average 6–14 km in a single one‐way trip (Dunn et al., 2008; Harrison, 2003; Jandér et al., 2016; Nason et al., 1998). Most fig wasp species that pollinate large rainforest trees disperse passively with the wind above the canopy during the day, exposing them to full sunlight and daytime temperatures (Harrison, 2003; Nason et al., 1998; Ware & Compton, 1994). Additionally, as tropical insects, they are expected to be more sensitive to temperature increases because they are likely to already perform close to their thermal maximum (Sunday et al., 2014). During their larval development, fig wasps are sheltered within fig fruits that, at least in some species, are actively cooled by the tree (Patiño et al., 1994). However, when the adult wasps leave their fig fruit to carry pollen and eggs to a new fig tree, wasps are exposed to the ambient temperature. Earlier studies of four Old world fig wasp genera (*Ceratosolen*, *Eupristina*, *Pleistodontes*, and *Valisia*) found that pollinator fig wasps can be extremely sensitive to increased temperatures resulting in dramatic decreases in lifespan (Aung et al., 2022; Gigante et al., 2020; Jevanandam et al., 2013; Sutton et al., 2018). Are these worrying findings generalizable also to New World pollinator fig wasps?

To fill this knowledge gap, we quantified the effect of increased air temperature on the lifespan of Neotropical fig wasps. Using climate chambers, we performed survival experiments on wasp species from both existing Neotropical fig pollinator genera, *Pegoscapus* and *Tetrapus*. Our tested wasp species are species‐specific pollinators of five common Panamanian *Ficus* species (but see Machado et al., 2005; Satler et al., 2020, regarding rare host sharing). We tested temperatures that ranged from the local current mean daytime temperatures to the local predicted daytime temperatures by the end of the 21st century (1–4°C higher depending on the different climate scenarios; IPCC, 2021) and beyond. Because our samples also contained some parasitic fig wasp genera, we opportunistically included also them in the study. If parasitic fig wasps respond differently than pollinators to increasing temperatures, the wasp community structure may change (Aung et al., 2022; Kordas et al., 2011). Specifically, we asked how increased air temperature affects the lifespan of (1) pollinator fig wasps and (2) parasitic fig wasps.

## 2 MATERIAL AND METHODS

### 2.1 Study site

**FIGURE 2:**
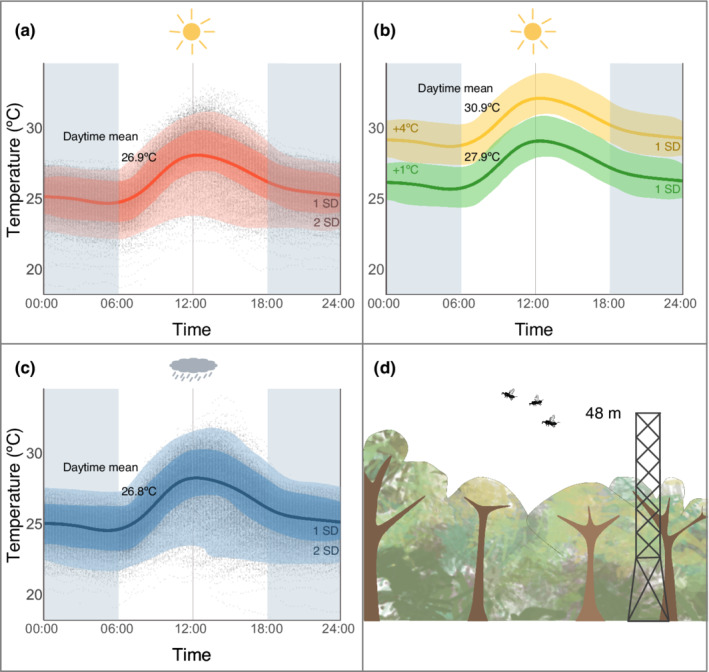
Current and projected temperatures above the canopy at Barro Colorado Island. Temperatures were measured every 15 min at 48 m above the ground in the forest of Barro Colorado Island, Panama (Paton, 2020). The mean ± 1 SD and 2 SD are indicated. (a) Data for the dry seasons 2002–2017, (b) projected dry season mean temperature ± 1 SD under local predictions for 2100 (best and worst climate scenario; IPCC, 2021), (c) data for the wet seasons 2002–2017, (d) the measurement station on the Lutz‐tower at 48 m is above the 40 m canopy, and at a relevant height for dispersing fig wasps (Harrison, 2003; Hubbell et al., 2014).

The experiments were performed at the Smithsonian Tropical Research Institute's field station Barro Colorado Island (BCI) in central Panama, during the dry season in January–March in the years 2015, 2016, and 2017. The local climate is quite stable with daytime mean temperatures above the canopy of 26.9°C (2SD: 22.1–31.1°C) in the dry season and 26.8°C (2SD: 21.6–31.7°C) in wet season (Figure 2; Paton, 2020). All wasp individuals were collected within Barro Colorado Nature Monument.

### 2.2 Study species

We quantified the lifespan of the fig wasps pollinating five common Panamanian fig species, representing the two genera of pollinator fig wasps present in the Neotropics: *Pegoscapus* (pollinators of *Ficus* subgenus Urostigma, subsection Americana) and *Tetrapus* (pollinators of *Ficus* subgenus Pharmacosycea, section Pharmacosycea) (Croat, 1978; Cruaud et al., 2012). Specifically, we studied *Pegoscapus tonduzi* (*Ficus citrifolia*), *P. hoffmeyeri* A and B (*F. obtusifolia*), *P. gemellus* A and B (*F. popenoei*), *Tetrapus costaricanus* (*F. insipida*), and *T. americanus* (*F. maxima*) (Molbo et al., 2004; Wiebes, 1995). *F. obtusifolia* and *F. popenoei* are each pollinated by two cryptic fig wasp species that cannot be distinguished morphologically (Molbo et al., 2004; Satler et al., 2020). For each fig species, one of the cryptic pollinator species is much more common than the other, and the wasps are sister species (*P. hoffmeyeri* A and B) or closely related (*P. gemellus* A and B) (Molbo et al., 2004; Satler et al., 2020) so in this study, we did not try to separate them. Non‐pollinating parasitic wasps were found on all fig species but only in two fig species were there a sufficient number of parasitic wasps in our samples to include them in the statistical analyses; *F. popenoei*: *Heterandrium* spp. and *Idarnes* “sensu stricto” (*Idarnes carme* spp. and *Idarnes flavicollis* spp. were combined and included; *Idarnes incerta* spp. were not included); and *F. insipida*: *Critogaster* spp. (Bouček, 1993; Cruaud et al., 2011; West & Herre, 1994) (see Appendix 1, Tables A1, A2, A3 for an overview of the study species and sample sizes). Parasitic wasps were identified to genus level (Bouček, 1993). Lifespans and temperature responses of the different species within each genus might differ, so the results for the parasitic wasps should be interpreted with caution. Additional information about the biology of the parasitic wasp genera is included in Appendix 1, part A4.

### 2.3 Survival experiment

To test the fitness effect of a temperature increase we quantified the lifespan of the wasps at 26, 28, 30, 32, 34, and 36°C (except for *P. hoffmeyeri* [*F. obtusifolia*] where the highest temperature tested was 34°C). We selected 26°C as the baseline temperature in the survival models because the mean diurnal temperature of the air above the canopy at BCI is around 26°C (Paton, 2020). The experimental temperatures 26, 28, and 30°C fall within one standard deviation of the current diurnal temperatures (Figure 2). The experimental temperatures 32, 34, and 36°C reflect the regionally projected temperature increase scenarios of 1–4°C by the end of the 21st century (IPCC, 2021). The daytime relative humidity of the air above the canopy at BCI ranges between 83% and 94% with higher values during the wet season (Paton, 2020). In our survival experiments, we used a constant relative humidity of 85% to mimic local natural conditions, and because low relative humidity can decrease wasp lifespan (Dunn et al., 2008).

To obtain the wasps used in the survival experiments, figs containing wasps were collected at dawn, within a few hours of when the wasps would naturally emerge from their figs. Figs were opened in the lab and wasps allowed to emerge into petri dishes; one petri dish per fig. To ensure that the wasps in the experiments were of similar age, we included only figs that upon opening were at the stage where several male wasps, but fewer than 20 female wasps, had emerged from their galls. Wasps were allowed to emerge for 2 h; we then removed the fig from the petri dish so that no further wasps would be added to the cohort of wasps. The number of wasps in each petri dish was on average 84.6 (range 34.5–149.8); full details in Appendix 1, Table A2. We aimed to test wasp cohorts originating from 20 independent figs per temperature treatment per fig species, but in some species (*F. obtusifolia*, *F. insipida*) we were restricted by the number of figs that were available at the correct developmental stage. In total, we tested wasps from 122 (*F. citrifolia*), 56 (*F. obtusifolia*), 120 (*F. popenoei*), 64 (*F. insipida*), and 116 (*F. maxima*) figs (Table A3). The petri dishes were sealed and kept in growth chambers (*Percival I‐36LL* and *Percival Intellus*) that mimicked natural environmental conditions for the wasps (12‐h light/dark regime and a relative humidity of 85%) (Paton, 2020). The only thing that differed between the different treatments was the temperature. To further ensure identical conditions, temperature and humidity were confirmed using an independent thermometer and hygrometer that were regularly transferred between the chambers.

The number of dead wasps was counted approximately every 4 h for the duration of the experiment. When death rate was very high, wasps were counted every 2 h or even every hour (in *F. insipida* 36°C, *F. popenoei* 28, 32, 34, 36°C, and *F. maxima* 34, 36°C) in order to capture the shape of all survival curves. A wasp was considered dead when it did not move, even after gentle tapping of the petri dish. The experiment ended when either all wasps were dead or all pollinator wasps were dead, except in *F. maxima* 28°C where five pollinator wasps remained alive at the end of the experiment, and *F. obtusifolia* 28°C (48 pollinators remained alive), 30°C (108 pollinators remained alive), and 32°C (eight pollinators remained alive). These were included in the analysis as right‐censored individuals.

### 2.4 Statistical analyses

To visualize differences in lifespan across treatments, survival curves for each treatment and wasp species were computed using packages “survival” (ver. 3.1‐12; Therneau & Lumley, 2020) and “survminer” (ver. 0.4.6; Kassambara et al., 2019) in R ver. 4.1.0 (R Core Team, 2021). To obtain the median lifespan across temperature treatments, the median lifespan duration and the 95% confidence interval were obtained using Kaplan–Meier survival analysis (package survival; function survfit and surv_median). We used a mixed effects Cox model (package “coxme”; Therneau, 2018) to model the effect of the temperature treatment on the survival probability of each species individually. The individual wasps inside each petri dish were not independent from each other because they shared their developmental environment (the fig) and were additionally potentially full sisters. We controlled for the variation between figs by including petri dish ID as a random factor in the survival model. We checked for all species if the random factor was significant by comparing the model with and without the random factor in an ANOVA; for all species, the random factor was significant (*p* < .001) except for *Heterandrium* (*p* = .97). The random factor was nevertheless included in all final models, as it corresponds to the experimental design. In the models for *Pegoscapus hoffmeyeri*, *Tetrapus costaricanus*, *Pegoscapus gemellus*, and *Heterandrium*, the highest temperature treatment (34°C resp. 36°C) had to be excluded from the analysis because there was not sufficient variation within these treatments. In the survival models, we used 26°C as the baseline temperature as this is the temperature closest to the mean diurnal temperature except for the model of *Pegoscapus tonduzi* where a baseline temperature of 28°C was used due to a lack of enough variance in the 26°C treatment. Additionally, significant differences in lifespan between all pairwise temperature comparisons were investigated using Tukey contrasts with Bonferroni adjusted *p*‐values, as implemented in the “multcomp” package (Bretz et al., 2010).

## 3 RESULTS

### 3.1 Pollinator response to temperature increase

**FIGURE 3:**
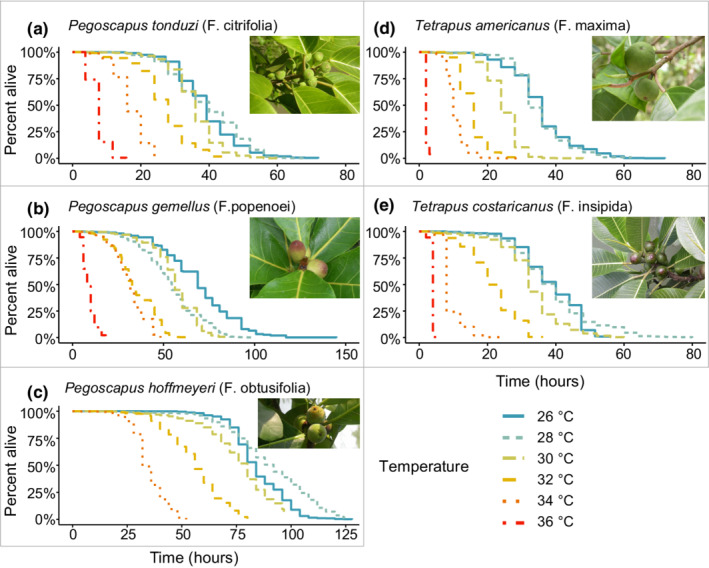
The lifespan of fig wasp pollinators of five common Panamanian fig tree species was dramatically shortened at higher temperatures. The baseline temperature of 26°C reflects the mean diurnal temperature above the canopy in years 2002–2017 (Paton, 2020).

**FIGURE 4:**
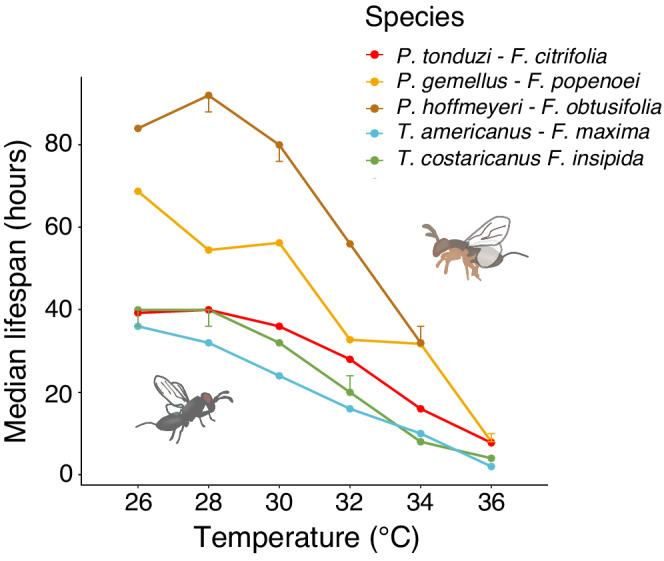
The median lifespan of pollinator fig wasps decreased with increasing temperatures (Kaplan–Meier survival analysis). Error bars represent 95% confidence intervals. Whereas lifespan maximum was either at 26 or 28°C for the different species, all tested pollinator fig wasps had significantly reduced median lifespans at 30°C and above.

For all pollinator species, we found a significant effect of temperature on lifespan (*P. tonduzi*: $\chi_{5}^{2}$ = 14,709, *p* < .001, *P. gemellus*: $\chi_{4}^{2}$ = 8109.5, *p* < .001, *P. hoffmeyeri*: $\chi_{5}^{2}$ = 381, *p* < .001, *T. americanus*: $\chi_{5}^{2}$ = 36,528, *p* < .001, *T. costaricanus*: $\chi_{4}^{2}$ = 2731, *p* < .001). All tested pollinator wasp species had a significantly shorter lifespan in 30, 32, 34, and 36°C compared to the baseline 26°C (*p* < .05; Figure 3, Table 1; Appendix 1, Tables A5, A6, A7, A8, A9). All pollinator species except *T. costaricanus* and *P. hoffmeyeri* also had a significantly shorter lifespan in 28°C compared to 26°C (*p* < .05; Figure 3, Table 1; Appendix 1, Tables A5, A6, A7, A8, A9), although the difference was no longer significant for *T. americanus* when testing all possible contrasts with Bonferroni adjusted *p*‐values (Appendix 1, Table A8). The reduction in lifespan was stronger in higher temperatures. For example, whereas the median lifespan of *P. tonduzi* (*F. citrifolia*) was 39 h at 26°C, it was only 28 h (71%) at 32°C, 16 h (41%) at 34°C, and 7.8 h (20%) at 36°C (Figure 4). At 26°C, the median lifespan of pollinator fig wasps ranged from 36 to 84 h depending on species, whereas at 36°C it ranged from 2 to 10 h (Figure 4). For some species (*Pegoscapus hoffmeyeri*, *Tetrapus costaricanus*, *Pegoscapus gemellus*), the highest temperature resulted in such a short lifespan that the reduced variation made it impossible to statistically model survival; these temperatures were therefore excluded from the final models.

**TABLE 1:** Results of the mixed effects cox model, comparing survival of each pollinator species at the different temperature treatments with the baseline treatment of 26°C.

	Treatment (°C)	coef	exp(coef) (±SE)	*z*	*p*
***Pegoscapus tonduzi*** *Ficus citrifolia* Events = 6744 *n* = 6744	26	0.46	1.59e+00 (±0.20)	2.27	.023
	30	0.81	2.25e+00 (±0.16)	5.22	<.001
	32	3.23	2.52e+01 (±0.16)	20.61	<.001
	34	6.00	4.05e+02 (±0.13)	45.86	<.001
	36	10.84	5.10e+04 (±0.17)	64.70	<.001
***Pegoscapus gemellus*** *Ficus popenoei* Events = 6524 *n* = 6524	28	1.44	4.23e+00 (±0.16)	9.25	<.001
	30	1.28	3.58e+00 (±0.13)	10.01	<.001
	32	4.48	8.83e+01 (±0.20)	22.28	<.001
	34	4.60	9.97e+01 (±0.14)	33.00	<.001
***Pegoscapus hoffmeyeri*** *Ficus obtusifolia* Events = 7439 *n* = 7603	28	−0.32	7.29e‐01 (±0.45)	−0.70	.480
	30	1.32	3.73e+00 (±0.48)	2.72	.007
	32	3.46	3.18e+01 (±0.47)	7.39	<.001
***Tetrapus americanus*** *Ficus maxima* Events = 14,259 *n* = 14,264	28	0.29	1.33e+00 (±0.13)	2.20	.028
	30	2.67	1.44e+01 (±0.09)	30.89	<.001
	32	5.03	1.53e+02 (±0.11)	48.06	<.001
	34	7.23	1.38e+03 (±0.10)	71.67	<.001
	36	14.72	2.46e+06 (±0.22)	67.11	<.001
***Tetrapus costaricanus*** *Ficus insipida* Events = 1999 *n* = 1999	28	−0.11	8.93e‐01 (±0.50)	−0.22	.820
	30	0.98	2.67e+00 (±0.39)	2.52	.012
	32	2.59	1.33e+01 (±0.40)	6.46	<.001
	34	5.42	2.27e+02 (±0.52)	10.48	<.001

*Note*: For *Pegoscapus tonduzi* a baseline of 28°C was used instead of 26°C. Coefficient is the estimated logarithm of hazard ratio, exponential (coef) transforms the log hazard ratio to hazard ratio between the compared treatments.

### 3.2 Parasite response to temperature increase

**FIGURE 5:**
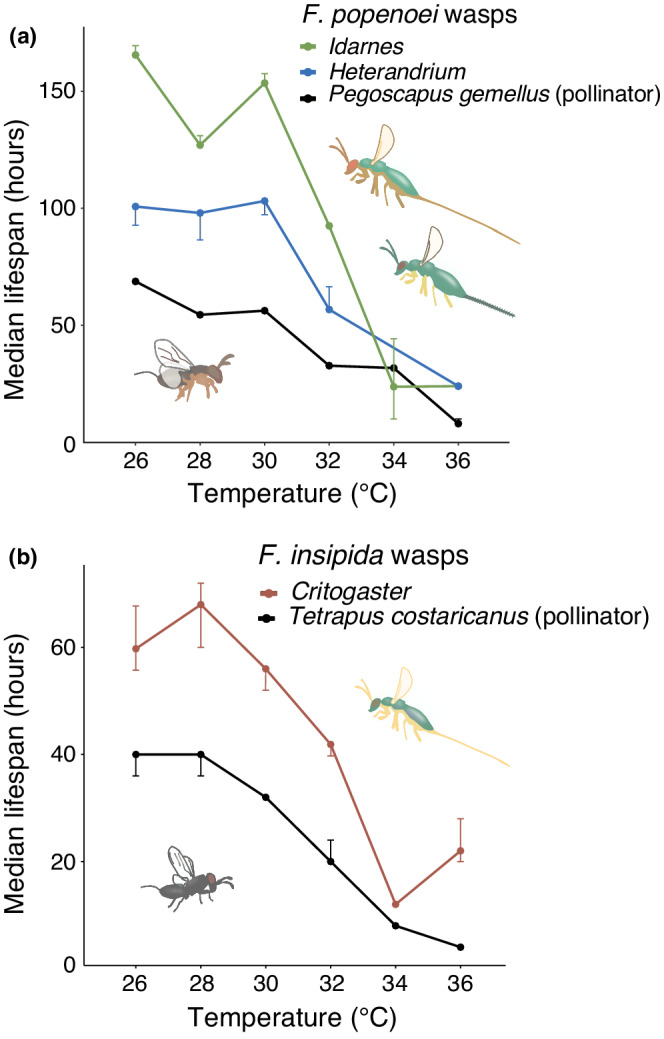
The median lifespan of pollinators and parasites of (a) *F. popenoei* and (b) *F. insipida* decreased with increasing temperatures (Kaplan–Meier survival analysis). Error bars represent 95% confidence intervals. Whereas maximum lifespan was either at 26, 28, or 30°C for the different species/genera, all tested parasitic wasps had significantly reduced median lifespans at 32°C and above. Wasp images are approximations for illustrative purposes.

Due to a much smaller sample size of parasitic wasps, we could only include three parasitic wasp genera, from a total of two fig species, in the analyses: *Idarnes* (*F. popenoei*), *Heterandrium* (*F. popenoei*), and *Critogaster* (*F. insipida*). As for pollinators, increased temperatures dramatically reduced the lifespan of these parasitic wasps (Figure 5, Table 2; Appendix 1, Tables A10, A11, A12). All parasitic genera had significantly shorter lifespan at temperatures higher than 30°C compared to at 26°C (Table 2; Appendix 1, Tables A10, A11, A12). For example, the median lifespan of *Idarnes* decreased from 165.5 h at 26°C to only 24 h (15%) at 36°C (Figure 5). *Idarnes* had significantly shorter lifespan already at 28°C, whereas *Heterandrium* and *Critogaster* did not have a significant lifespan reduction until 32°C (Figure 5, Table 2). In all cases, the parasitic wasps on average lived longer than the pollinators of their respective host fig species (Figure 5).

**TABLE 2:** Results of the mixed effects cox model, comparing survival of each parasitic species at the different temperature treatments with the baseline treatment of 26°C.

	Treatment (°C)	coef	exp(coef) (±SE)	*z*	*p*
***Idarnes carme* spp**. **and *Idarnes flavicollis* spp.** *Ficus popenoei* Events = 3750 *n* = 4220	28	2.503	1.22e+01 (±0.16)	15.73	**<.001**
	30	1.11	3.02e+00 (±0.20)	5.51	**<.001**
	32	4.51	9.13e+01 (±0.21)	21.51	**<.001**
	34	9.34	1.14e+04 (±0.49)	18.90	**<.001**
	36	10.62	4.07e+04 (±0.35)	30.15	**<.001**
***Heterandrium* spp.** *Ficus popenoei* Events = 40 *n* = 40	28	0.39	1.48e+00 (±0.56)	0.69	.490
	30	0.30	1.34e+00 (±0.85)	0.35	.730
	32	2.49	1.21e+02 (±0.64)	3.89	**<.001**
***Critogaster* spp.** *Ficus insipida* Events = 244 *n* = 262	28	−0.96	0.38e+00 (±0.58)	−1.67	.096
	30	0.45	1.57e+00 (±0.63)	0.72	.470
	32	2.61	1.36e+01 (±0.62)	4.24	**<.001**
	34	6.38	5.93e+02 (±0.86)	7.39	**<.001**
	36	4.83	1.25e+02 (±0.64)	7.58	**<.001**

*Note*: Coefficient is the estimated logarithm of hazard ratio, exponential (coef) transforms the log hazard ratio to hazard ratio between the compared treatments.

## 4 DISCUSSION

All studied fig wasp species showed a dramatic decrease in lifespan with increasing temperatures. Depending on the different CO_2_ emission scenarios, temperatures in Panama are expected to rise 1–4°C by the end of the 21st century (IPCC, 2021). A 4°C increase in temperature from the daytime mean 26–30°C reduced pollinator wasp median lifespan to 67% for some species. However, temperatures fluctuate during the day, and a 4°C increase could lead to daytime temperatures frequently reaching 33.8°C (1 SD above mean; Figure 2) (IPCC, 2021). This would severely impact fig pollinator lifespan, reducing the median lifespan to merely 20%–46% of the current lifespan.

Extreme weather events are also expected to increase under influence of global warming (IPCC, 2021; Kirtman et al., 2013). Exceptionally warm days, or even hours, would reduce wasps' lifespan dramatically. The median pollinator lifespan at 36°C was in our study reduced to merely 2–10 h (6%–19% of the baseline median lifespan), and in tropical Singapore to 1–4.5 h (4%–15% of baseline median lifespan) (Jevanandam et al., 2013). Pollinator fig wasps in temperate Australia, a more variable climate than the tropics, were slightly more tolerant to such high temperatures (50% median lifespan reduction at 35°C), but experienced a reduction to 14% median lifespan at 40°C (Sutton et al., 2018). Temperatures of 40°C already occur near the Australian study site approximately 5 days per year (Sutton et al., 2018). Extreme weather events such as these (36°C in tropics, 40°C in temperate regions) could both kill wasps while still inside the fig, and prevent dispersal of already emerged adults (Jevanandam et al., 2013; Sutton et al., 2018). Temperatures of 36°C are currently not occurring above the forest canopy in central Panama; the maximum measured temperature in 2002–2017 was 34.6°C (Paton, 2020). However, with a projected temperature increase of 4°C, temperatures above 36°C are expected to occur more frequently.

While all pollinator species showed a clear reduction in lifespan with increased temperatures, the reduction was particularly dramatic for those wasp species that had higher median lifespans at the baseline temperature 26°C (Figure 5). Although we here have tested too few species to reliably compare across species, a trend seems to be that wasps of the genus *Pegoscapus* (pollinating fig trees of section Urostigma Americana) have higher median lifespans than wasps of the genus *Tetrapus* (pollinating fig trees of section Pharmacosycea). This may reflect density of the host fig species in a natural forest, but we currently do not have sufficient data to test this hypothesis. Within a genus, larger wasp species seem to have longer median lifespans than smaller wasp species (Herre, 1989; Jandér et al., 2016) but additional species would be needed to test this hypothesis.

The parasitic wasps also showed a clear reduction in lifespan with increasing temperatures (Figure 5). The parasitic wasp genera we tested had a 47%–141% higher median lifespan at baseline temperature than did the pollinator of their host fig species, despite being of similar body sizes as the pollinator. At 34°C, parasitic wasp lifespan was reduced to 14%–20%. Because the parasitic wasp genera and pollinators we tested were affected similarly by increasing temperatures, our data do not suggest that temperature increase per se would be expected to lead to a dramatic change in fig wasp community composition. However, our lifespan data for the parasitic wasps should be interpreted with caution. We grouped the parasitic wasps according to genus, but the individual species within a genus might respond differently to increased temperatures. Also, due to practicalities of the experimental setup, we were unable to feed the wasps during the lifespan assays. This is not an issue for pollinators as they are known to not eat, but some parasitic fig wasp genera are known to have extended lifespans if food (sucrose solution) is offered (Compton et al., 1994; Ghara & Borges, 2010). However, the difference in lifespan with and without food might not be dramatic. Van Goor et al. (2018, 2021) found a median lifespan of 168 h when offering sugar water to the parasitic wasps *Idarnes flavicollis* ssp. of *Ficus petiolaris* at 22°C (J. van Goor personal communication, 2021). Additionally, a pilot study by Van Goor in Panama found median lifespans of *Idarnes carme* ssp. and *Idarnes flavicollis* ssp. of *F. popenoei*, at ambient temperature with food, to be 100–130 h, compared to the median lifespan of 166 h we found for *Idarnes* of *F. popenoei* at 26°C without food (J. Van Goor personal communication, 2021; Figure 5). Although our methods differ, therefore making direct comparison impossible, it seems that access to food may not dramatically prolong life for *Idarnes*, and our lifespan estimates for these parasites may be valid approximations.

A reduced lifespan of pollinator wasps is expected to reduce pollination levels and pollen dispersal of fig trees. Despite their short lives (2–3 days), fig wasps disperse large distances because fig trees typically grow in low densities, even for tropical trees (Mawdsley et al., 1998; Nason et al., 1996, 1998; Todzia, 1986; Ware & Compton, 1994). For example, in central Panama fig wasps commonly disperse 10 km, but on other continents occasional dispersal distances up to 160 km have been recorded (Ahmed et al., 2009; Nason et al., 1996). If fewer fig wasps successfully disperse to flowering trees, fewer of the figs will be pollinated thus producing fewer fruits for the frugivores (Herre, 1989; Jandér et al., 2016; Shanahan et al., 2001). Additionally, each fig inflorescence is likely to be pollinated by fewer foundress fig wasps, thus reducing overall seed numbers within each fig (Herre, 1989; Jandér et al., 2012; Jandér & Steidinger, 2017) and increasing inbreeding of the wasps (Herre, 1985; Herre et al., 2008; Molbo et al., 2004). Further adding to the problem, logging practices that reduce South American forest area with 2.6 million hectares per year cause forest fragmentation, thus further increasing distances between fig trees (Broadbent et al., 2008; FAO, 2020; Hansen et al., 2013; Mawdsley et al., 1998). Logging also increases the local temperature by biomass removal—the air above logged areas can be 5–10°C hotter than nearby intact forest environment (Blonder et al., 2018). Forest fragmentation therefore not only increases distances between trees, but might also reduce wasp lifespan even further. Forest fragmentation in combination with global warming could therefore be devastating for the continued pollination of fig trees.

Highly specialized and obligate mutualisms are expected to be more vulnerable to the effects of rapid environmental change than relationships based on more generalist partners (Kiers et al., 2010; Vidal et al., 2021). In highly obligate mutualisms like the fig tree—fig wasp mutualisms, behavioral changes, plasticity, or rapid adaptation may be essential for the continuation of the mutualism. Behavioral changes of the pollinator fig wasps would be the fastest response, for example by emerging from figs earlier in the morning or by dispersing during the night like some species in Australia and East‐Asia (Harrison, 2003). However, because wind speeds are lower at night, this would likely compromise wasps' dispersal distances, and therefore the success rate of finding a flowering tree (Harrison & Rasplus, 2006; Kumagai et al., 2001; Paton, 2020). Rapid adaptation might be a possibility, favored by the short generation time of the wasps. However, several of these pollinator fig wasp species are highly inbred due to frequent sibling matings (inbreeding coefficient *F* up to 0.85; Molbo et al., 2004), reducing genetic variability. Nevertheless, in our experiments, as in Jevanandam et al. (2013), there were differences in wasp lifespan across different wasp sibling groups (wasp cohorts emerging from the same fig). This variation could be caused by either environmental or genetic factors, or a combination. If sufficient genetic variation is present, adaptation can be fast: studies of temperate mosquitoes showed that adaptation to heat can occur within as few as three generations (Foucault et al., 2018). However, tropical insects may be less capable of adapting as many already live at their thermal maxima, and ectotherm thermal limits seem to evolve at a rate of only 0.8°C per million years (Bennett et al., 2021; García‐Robledo & Baer, 2021; Sunday et al., 2014). Further studies on the possibility of tropical pollinators' adaptation to increasing temperatures would be valuable.

In conclusion, the projected local temperature increases in Panama could seriously decrease the lifespan of fig tree pollinators. By reducing the lifespan of fig wasps and therefore the chances of successful pollination of fig trees, increasing temperatures add an additional threat to this keystone resource of tropical forests. Anthropogenic ecosystem changes in the form of habitat destruction, fragmentation, and temperature increases, require species to cope with new situations. Particular attention should be paid to species in tight mutualistic relationships as they are vulnerable also to effects on their mutualistic partners.

## AUTHOR CONTRIBUTIONS

**Lisette van Kolfschoten:** Formal analysis (equal); visualization (lead); writing – original draft (lead); writing – review and editing (lead). **Lovisa Dück:** Investigation (lead); writing – review and editing (supporting). **Martin I. Lind:** Formal analysis (equal); writing – review and editing (supporting). **K. Charlotte Jandér:** Conceptualization (lead); formal analysis (supporting); funding acquisition (lead); investigation (supporting); methodology (lead); supervision (lead); writing – original draft (supporting); writing – review and editing (lead).

## CONFLICT OF INTEREST

All authors hereby state that we have no competing interests to declare.

## Data Availability

Wasp lifespan data and R code are available on FigShare (DOI: https://doi.org/10.6084/m9.figshare.20268552.v2).

## APPENDIX 1

**TABLE A1:** Overview of the different species included in this study.

Tree	Tree species	Pollinator	Parasite
Subgenus *Urostigma* Section *Americana*	*Ficus citrifolia* (Molbo et al., 2004; Wiebes, 1995)	*Pegoscapus tonduzi*	*Idarnes*
	*Ficus obtusifolia* (Molbo et al., 2004; Satler et al., 2020; Wiebes, 1995)	*Pegoscapus hoffmeyeri A* *Pegoscapus hoffmeyeri B*	*Idarnes*
	*Ficus popenoei* (Molbo et al., 2004; Satler et al., 2020; Wiebes, 1995)	*Pegoscapus gemellus* A *Pegoscapus gemellus* B	*Idarnes*, *Heterandrium*
Subgenus *Pharmacosycea* Section *Pharmacosycea*	*Ficus insipida* (Molbo et al., 2004; Wiebes, 1995)	*Tetrapus costaricanus*	*Critogaster*
	*Ficus maxima* (Molbo et al., 2004; Wiebes, 1995)	*Tetrapus americanus*	*Critogaster*

**TABLE A2:** Summary of the overall sample sizes in this study. See Table A3 for the number of figs (i.e., wasp cohorts) and individual wasps in each temperature treatment.

Tree species	Figs (*n*)	Pollinator wasps (*n*)	Mean number of pollinator wasps per fig (range)	Parasitic wasps (*n*)
*Ficus citrifolia*	122	6744	55.28 (18–153)	1
*Ficus obtusifolia*	56	8391	149.84 (12–365)	6
*Ficus popenoei*	120	7260	60.50 (6–271)	4287
*Ficus insipida*	64	2208	34.50 (2–190)	262
*Ficus maxima*	116	14,264	122.97 (28–234)	159

*Note*: The number of parasitic wasps indicated here are those of the genera *Idarnes*, *Heterandrium*, and *Critogaster*; additional parasitic wasps genera were present but not included in the study.

**TABLE A3:** Number of figs (ie wasp cohorts) and individual wasps included in the analyses.

Tree species	Sample size (*n*)	26°C	28°C	30°C	32°C	34°C	36°C	Total
*Ficus citrifolia*	Figs	20	20	20	20	20	22	122
	Pollinators	957	1373	1342	852	1101	1120	6744
*Ficus obtusifolia*	Figs	10	15	11	13	15		56
	Pollinators	1140	3740	1540	1183	788		8391
*Ficus popenoei*	Figs	20	20	20	21	20	15	120
	Pollinators	1399	2813	1483	2341	1282	1881	7260
	Parasites *Idarnes*	320	472	730	1576	15	1107	4220
	Parasites *Heterandrium*	6	9	2	23	0	27	67
*Ficus insipida*	Figs	10	5	13	12	5	11	64
	Pollinators	440	360	576	496	293	318	2208
	Parasites *Critogaster*	40	54	31	28	7	104	262
*Ficus maxima*	Figs	20	20	20	20	20	20	116
	Pollinators	2146	2871	2623	1612	2330	2681	14,264

### A4. Biology of the parasitic wasp genera

Parasitic fig wasps, also referred to as non‐pollinating fig wasps (NPFW), are wasp species that obligately rely on figs for their reproduction but do not provide any pollination services to the fig (Bouček, 1993; Bronstein, 1991; Marussich & Machado, 2007; West & Herre, 1994). New world parasitic fig wasps all oviposit from the external surface of the fig wall to reach the flowers or other tissues within (West et al., 1996). *Idarnes carme* spp., *Idarnes flavicollis* spp., and *Critogaster* spp. are in direct competition with the pollinator wasps over available flowers (Bronstein, 1991; Canesqui da Costa & Graciolli, 2010; Elias et al., 2008; Gordh, 1975; Marussich & Machado, 2007; Santinelo Pereira et al., 2007; West et al., 1996). *Idarnes carme* spp. lay their eggs in galls with developing pollinator wasps inside (Elias et al., 2008), whereas *Idarnes flavicollis* spp. gall the flowers (Elias et al., 2012). In contrast, *Heterandrium* spp. lay their eggs directly into the fig wall where they form galls (Bronstein, 1991; Elias et al., 2008; West et al., 1996). Parasitic fig wasps are generally less species specific than the pollinator wasps (Farache et al., 2018; Marussich & Machado, 2007).

**TABLE A5:** Multiple comparisons of all pairwise temperature treatments for the pollinator *Pegoscapus tonduzi* in *Ficus citrifolia*, using Tukey contrasts with Bonferroni adjusted *p*‐values.

Contrast	Estimate	SE	*z*	*p*
26°C – 28°C	0.461	0.203	2.28	.193
30°C – 28°C	0.809	0.155	5.22	**<.001**
32°C – 28°C	3.227	0.157	20.61	**<.001**
34°C – 28°C	6.004	0.131	45.86	**<.001**
36°C – 28°C	10.839	0.168	64.70	**<.001**
30°C – 26°C	0.348	0.216	1.62	.570
32°C – 26°C	2.766	0.178	15.57	**<.001**
34°C – 26°C	5.544	0.166	33.39	**<.001**
36°C – 26°C	10.379	0.196	52.86	**<.001**
32°C – 30°C	2.418	0.169	14.35	**<.001**
34°C – 30°C	5.195	0.134	38.78	**<.001**
36°C – 30°C	10.031	0.173	58.01	**<.001**
34°C – 32°C	2.778	0.133	20.93	**<.001**
36°C – 32°C	7.613	0.160	47.63	**<.001**
36°C – 34°C	4.835	0.124	38.98	**<.001**

*Note*: Significant contrasts are indicated in bold.

**TABLE A6:** Multiple comparisons of all pairwise temperature treatments for the pollinator *Pegoscapus gemellus* in *Ficus popenoei* using Tukey contrasts with Bonferroni adjusted *p*‐values.

Contrast	Estimate	SE	*z*	*p*
28°C – 26°C	1.443	0.156	9.25	**<.001**
30°C – 26°C	1.275	0.127	10.01	**<.001**
32°C – 26°C	4.481	0.201	22.28	**<.001**
34°C – 26°C	4.602	0.139	33.01	**<.001**
30°C – 28°C	−0.168	0.155	−1.09	.793
32°C – 28°C	3.038	0.214	14.21	**<.001**
34°C – 28°C	3.159	0.152	20.77	**<.001**
32°C – 30°C	3.206	0.200	16.03	**<.001**
34°C – 30°C	3.327	0.127	26.12	**<.001**
34°C – 32°C	0.122	0.255	0.48	.988

*Note*: Significant contrasts are indicated in bold.

**TABLE A7:** Multiple comparisons of all pairwise temperature treatments for the pollinator *Pegoscapus hoffmeyeri* in *Ficus obtusifolia* using Tukey contrasts with Bonferroni adjusted *p*‐values.

Contrast	Estimate	SE	*z*	*p*
28°C – 26°C	−0.317	0.451	−0.70	.896
30°C – 26°C	1.316	0.484	2.72	**.034**
32°C – 26°C	3.458	0.468	7.39	**<.001**
30°C – 28°C	1.633	0.440	3.72	**.001**
32°C – 28°C	3.775	0.421	8.96	**<.001**
32°C – 30°C	2.142	0.456	4.70	**<.001**

*Note*: Significant contrasts are indicated in bold.

**TABLE A8:** Multiple comparisons of all pairwise temperature treatments for the pollinator *Tetrapus americanus* in *Ficus maxima* using Tukey contrasts with Bonferroni adjusted *p*‐values.

Contrast	Estimate	SE	*z*	*p*
28°C – 26°C	0.288	0.131	2.20	.210
30°C – 26°C	2.668	0.086	30.89	**<.001**
32°C – 26°C	5.032	0.105	48.06	**<.001**
34°C – 26°C	7.230	0.101	71.67	**<.001**
36°C – 26°C	14.717	0.219	67.11	**<.001**
30°C – 28°C	2.380	0.097	24.54	**<.001**
32°C – 28°C	4.743	0.090	52.70	**<.001**
34°C – 28°C	6.942	0.095	73.27	**<.001**
36°C – 28°C	14.429	0.216	66.73	**<.001**
32°C – 30°C	2.364	0.088	26.86	**<.001**
34°C – 30°C	4.562	0.073	62.15	**<.001**
36°C – 30°C	12.049	0.208	57.81	**<.001**
34°C – 32°C	2.199	0.085	25.93	**<.001**
36°C – 32°C	9.686	0.210	46.09	**<.001**
36°C – 34°C	7.487	0.198	37.80	**<.001**

*Note*: Significant contrasts are indicated in bold.

**TABLE A9:** Multiple comparisons of all pairwise temperature treatments for the pollinator *Tetrapus costaricanus* in *Ficus insipida* using Tukey contrasts with Bonferroni adjusted *p*‐values.

Contrast	Estimate	SE	*z*	*p*
28°C – 26°C	−0.114	0.507	−0.22	.999
30°C – 26°C	0.982	0.391	2.52	.085
32°C – 26°C	2.585	0.400	6.46	**<.001**
34°C – 26°C	5.424	0.518	10.48	**<.001**
30°C – 28°C	1.096	0.488	2.25	.159
32°C – 28°C	2.698	0.496	5.44	**<.001**
34°C – 28°C	5.537	0.595	9.31	**<.001**
32°C – 30°C	1.603	0.372	4.30	**<.001**
34°C – 30°C	4.442	0.496	8.96	**<.001**
34°C – 32°C	2.839	0.498	5.70	**<.001**

*Note*: Significant contrasts are indicated in bold.

**TABLE A10:** Multiple comparisons of all pairwise temperature treatments for the parasitic wasps *Idarnes carme* spp. and *Idarnes flavicollis* spp. in *Ficus popenoei* using Tukey contrasts with Bonferroni adjusted *p*‐values.

Contrast	Estimate	SE	*z*	*p*
28°C – 26°C	2.503	0.159	15.73	**<.001**
30°C – 26°C	1.105	0.201	5.51	**<.001**
32°C – 26°C	4.514	0.210	21.51	**<.001**
34°C – 26°C	9.341	0.494	18.90	**<.001**
36°C – 26°C	10.615	0.352	30.15	**<.001**
30°C – 28°C	−1.398	0.185	−7.58	**<.001**
32°C – 28°C	2.011	0.186	10.82	**<.001**
34°C – 28°C	6.838	0.482	14.18	**<.001**
36°C – 28°C	8.111	0.335	24.21	**<.001**
32°C – 30°C	3.409	0.204	16.74	**<.001**
34°C – 30°C	8.236	0.493	16.72	**<.001**
36°C – 30°C	9.510	0.350	27.20	**<.001**
34°C – 32°C	4.827	0.489	9.87	**<.001**
36°C – 32°C	6.101	0.336	18.17	**<.001**
36°C – 34°C	1.273	0.536	2.38	.145

*Note*: Significant contrasts are indicated in bold.

**TABLE A11:** Multiple comparisons of all pairwise temperature treatments for the parasitic wasp *Heterandrium* spp*.* in *Ficus popenoei* using Tukey contrasts with Bonferroni adjusted *p*‐values.

Contrast	Estimate	SE	*z*	*p*
28°C – 26°C	0.390	0.561	0.70	.896
30°C – 26°C	0.295	0.851	0.35	.985
32°C – 26°C	2.494	0.641	3.89	**<.001**
30°C – 28°C	−0.095	0.797	−0.12	.999
32°C – 28°C	2.104	0.547	3.84	**<.001**
32°C – 30°C	2.199	0.829	2.65	**.038**

*Note*: Significant contrasts are indicated in bold.

**TABLE A12:** Multiple comparisons of all pairwise temperature treatments for the parasitic wasp *Critogaster* spp. in *Ficus insipida* using Tukey contrasts with Bonferroni adjusted *p*‐values.

Contrast	Estimate	SE	*z*	*p*
28°C – 26°C	−0.959	0.576	−1.67	.542
30°C – 26°C	0.448	0.627	0.72	.979
32°C – 26°C	2.608	0.615	4.24	**<.001**
34°C – 26°C	6.384	0.863	7.39	**<.001**
36°C – 26°C	4.827	0.637	7.58	**<.001**
30°C – 28°C	1.408	0.612	2.30	.185
32°C – 28°C	3.568	0.603	5.92	**<.001**
34°C – 28°C	7.344	0.855	8.59	**<.001**
36°C – 28°C	5.786	0.625	9.26	**<.001**
32°C – 30°C	2.160	0.593	3.64	**.003**
34°C – 30°C	5.936	0.846	7.02	**<.001**
36°C – 30°C	4.379	0.613	7.15	**<.001**
34°C – 32°C	3.776	0.762	4.95	**<.001**
36°C – 32°C	2.219	0.493	4.50	**<.001**
36°C – 34°C	−1.558	0.691	−2.25	.205

*Note*: Significant contrasts are indicated in bold.

## References

[ece39311-bib-0001] Ågren, J. (1996). Population size, pollinator limitation, and seed set in the self‐incompatible herb *Lythrum salicaria* . Ecology, 77, 1779–1790.

[ece39311-bib-0002] Ahmed, S. , Compton, S. G. , Butlin, R. K. , & Gilmartin, P. M. (2009). Wind‐borne insects mediate directional pollen transfer between desert fig trees 160 kilometers apart. Proceedings of the National Academy of Sciences of the United States of America, 106, 20342–20347. 19910534 10.1073/pnas.0902213106 PMC2787140

[ece39311-bib-0003] Angilletta, M. J., Jr. , & Angilletta, M. J. (2009). Thermal adaptation: A theoretical and empirical synthesis. Oxford Univeristy Press.

[ece39311-bib-0004] Aung, K. M. M. , Chen, H. H. , Segar, S. T. , Miao, B. G. , Peng, Y. Q. , & Liu, C. (2022). Changes in temperature alter competitive interactions and overall structure of fig wasp communities. Journal of Animal Ecology, 91, 1303–1315. 35420162 10.1111/1365-2656.13701

[ece39311-bib-0005] Bennett, J. M. , Sunday, J. , Calosi, P. , Villalobos, F. , Martínez, B. , Molina‐Venegas, R. , Araújo, M. B. , Algar, A. C. , Clusella‐Trullas, S. , Hawkins, B. A. , Keith, S. A. , Kühn, I. , Rahbek, C. , Rodríguez, L. , Singer, A. , Morales‐Castilla, I. , & Olalla‐Tárraga, M. Á. (2021). The evolution of critical thermal limits of life on earth. Nature Communications, 12, 1–9. 10.1038/s41467-021-21263-8 PMC7895938 33608528

[ece39311-bib-0006] Biesmeijer, J. C. (2006). Parallel declines in pollinators and insect‐pollinated plants in Britain and The Netherlands. Science, 313, 351–354. 16857940 10.1126/science.1127863

[ece39311-bib-0007] Blonder, B. , Both, S. , Coomes, D. A. , Elias, D. , Jucker, T. , Kvasnica, J. , Majalap, N. , Malhi, Y. S. , Milodowski, D. , Riutta, T. , & Svátek, M. (2018). Extreme and highly heterogeneous microclimates in selectively logged tropical forests. Frontiers in Forests and Global Change, 1, 1–14.

[ece39311-bib-0008] Bouček, Z. (1993). The genera of chalcidoid wasps from *ficus* fruit in the new world. Journal of Natural History, 27, 173–217.

[ece39311-bib-0009] Bretz, F. , Hothorn, T. , & Westfall, P. (2010). Multiple comparisons using R. Chapman and Hall/CRC.

[ece39311-bib-0010] Broadbent, E. N. , Asner, G. P. , Keller, M. , Knapp, D. E. , Oliveira, P. J. C. , & Silva, J. N. (2008). Forest fragmentation and edge effects from deforestation and selective logging in the Brazilian Amazon. Biological Conservation, 141, 1745–1757.

[ece39311-bib-0011] Bronstein, J. L. (1991). The nonpollinating wasp fauna of *Ficus pertusa*: Exploitation of a mutualism? Oikos, 61, 175–186.

[ece39311-bib-0012] Brosi, B. J. , & Briggs, H. M. (2013). Single pollinator species losses reduce floral fidelity and plant reproductive function. Proceedings of the National Academy of Sciences of the United States of America, 110, 13044–13048. 23878216 10.1073/pnas.1307438110 PMC3740839

[ece39311-bib-0013] Canesqui da Costa, P. , & Graciolli, G. (2010). Insects associated with syconia of *Ficus citrifolia* (Moraceae) in central Brazil. Revista Brasileira de Entomologia, 54, 707–709.

[ece39311-bib-0014] Compton, S. G. , Rasplus, J. Y. , & Ware, A. B. (1994). African fig wasp parasitoid communities. In B. Hawkins & W. Sheehan (Eds.), Parasitoid community ecology (pp. 343–368). Oxford Univeristy Press.

[ece39311-bib-0015] R Core Team . (2021). R: A language and environment for statistical computing. R Foundation for Statistical Computing.

[ece39311-bib-0016] Croat, T. B. (1978). Flora of Barro Colorado Island. Stanford University Press.

[ece39311-bib-0017] Cruaud, A. , Jabbour‐Zahab, R. , Genson, G. , Kjellberg, F. , Kobmoo, N. , Van Noort, S. , Da‐Rong, Y. , Yan‐Qiong, P. , Ubaidillah, R. , Hanson, P. E. , Santos‐Mattos, O. , Farache, F. H. A. , Pereira, R. A. S. , Kerdelhué, C. , & Rasplus, J. Y. (2011). Phylogeny and evolution of life‐history strategies in the Sycophaginae non‐pollinating fig wasps (Hymenoptera, Chalcidoidea). BMC Evolutionary Biology, 11, 178. 21696591 10.1186/1471-2148-11-178 PMC3145598

[ece39311-bib-0018] Cruaud, A. , Ronsted, N. , Chantarasuwan, B. , Chou, L. S. , Clement, W. L. , Couloux, A. , Cousins, B. , Genson, G. , Harrison, R. D. , Hanson, P. E. , Hossaert‐Mckey, M. , Jabbour‐Zahab, R. , Jousselin, E. , Kerdelhué, C. , Kjellberg, F. , Lopez‐Vaamonde, C. , Peebles, J. , Peng, Y. Q. , Santinelo Pereira, R. A. , … Savolainen, V. (2012). An extreme case of plant‐insect codiversification: Figs and fig‐pollinating wasps. Systematic Biology, 61, 1029–1047. 22848088 10.1093/sysbio/sys068 PMC3478567

[ece39311-bib-0019] Datwyler, S. L. , & Weiblen, G. D. (2004). On the origin of the fig: Phylogenetic relationships of Moraceae from NDHF sequences. American Journal of Botany, 91, 767–777. 21653431 10.3732/ajb.91.5.767

[ece39311-bib-0020] Dunn, D. W. , Yu, D. W. , Ridley, J. , & Cook, J. M. (2008). Longevity, early emergence and body size in a pollinating fig wasp ‐ Implications for stability in a fig‐pollinator mutualism. Journal of Animal Ecology, 77, 927–935. 18624736 10.1111/j.1365-2656.2008.01416.x

[ece39311-bib-0021] Durak, R. , Dampc, J. , & Dampc, J. (2020). Role of temperature on the interaction between Japanese quince *Chaenomeles japonica* and herbivorous insect *Aphis pomi* (Hemiptera: Aphidoidea). Environmental and Experimental Botany, 176, 104100.

[ece39311-bib-0022] Elias, L. G. , Menezes, A. O. , & Pereira, R. A. S. (2008). Colonization sequence of non‐pollinating fig wasps associated with *Ficus citrifolia* in Brazil. Symbiosis, 45, 107–111.

[ece39311-bib-0023] Elias, L. G. , Teixeira, S. P. , Kjellberg, F. , & Pereira, R. A. S. (2012). Diversification in the use of resources by Idarnes species: Bypassing functional constraints in the fig–fig wasp interaction. Biological Journal of the Linnean Society, 106(1), 114–122.

[ece39311-bib-0024] FAO . (2020). Global forest resources assessment 2020 – Key findings. Rome.

[ece39311-bib-0025] Farache, F. H. A. , Cruaud, A. , Rasplus, J. Y. , Cerezini, M. T. , Rattis, L. , Kjellberg, F. , & Pereira, R. A. S. (2018). Insights into the structure of plant‐insect communities: Specialism and generalism in a regional set of non‐pollinating fig wasp communities. Acta Oecologica, 90, 49–59.

[ece39311-bib-0026] Feder, M. E. , Blair, N. , & Figueras, H. (1997). Natural thermal stress and heat‐shock protein expression in *Drosophila* larvae and pupae. Functional Ecology, 11, 90–100.

[ece39311-bib-0027] Foucault, Q. , Wieser, A. , Waldvogel, A. M. , Feldmeyer, B. , & Pfenninger, M. (2018). Rapid adaptation to high temperatures in *Chironomus riparius* . Ecology and Evolution, 8, 12780–12789. 30619582 10.1002/ece3.4706 PMC6308882

[ece39311-bib-0028] García‐Robledo, C. , & Baer, C. S. (2021). Positive genetic covariance and limited thermal tolerance constrain tropical insect responses to global warming. Journal of Evolutionary Biology, 34, 1432–1446. 34265126 10.1111/jeb.13905

[ece39311-bib-0029] Ghara, M. , & Borges, R. M. (2010). Comparative life‐history traits in a fig wasp community: Implications for community structure. Ecological Entomology, 35, 139–148.

[ece39311-bib-0030] Gigante, E. T. , Lim, E. J. , Crisostomo, K. G. , Cornejo, P. , & Rodriguez, L. J. (2020). Increase in humidity widens heat tolerance range of tropical *Ceratosolen* fig wasps. Ecological Entomology, 46, 573–581.

[ece39311-bib-0031] Gordh, G. (1975). Comparative external morphology and systematics of the neotropical parasitic fig wasp genus Idarnes (Hymenoptera: Torymidae). University of Kansas Science Bulletin, 50, 389–455.

[ece39311-bib-0032] Hallmann, C. A. , Sorg, M. , Jongejans, E. , Siepel, H. , Hofland, N. , Sumser, H. , Ho, T. , Schwan, H. , Stenmans, W. , Mu, A. , Goulson, D. , & De Kroon, H. (2017). More than 75 percent decline over 27 years in total flying insect biomass in protected areas. PLoS One, 12, 1–21. 10.1371/journal.pone.0185809 PMC5646769 29045418

[ece39311-bib-0033] Hansen, M. C. , Potapov, P. V. , Moore, R. , Hancher, M. , Turubanova, S. A. , Tyukavina, A. , Thau, D. , Stehman, S. V. , Goetz, S. J. , Loveland, T. R. , Kommareddy, A. , Egorov, A. , Chini, L. , Justice, C. O. , & Townshend, J. R. G. (2013). High‐resolution global maps of 21st‐century forest cover change. Science, 342, 850–853. 24233722 10.1126/science.1244693

[ece39311-bib-0034] Harrison, R. D. (2003). Fig wasp dispersal and the stability of a keystone plant resource in Borneo. Proceedings of the Royal Society B: Biological Sciences, 270, 76–79. 10.1098/rsbl.2003.0018 PMC1698007 12952642

[ece39311-bib-0035] Harrison, R. D. , & Rasplus, J. Y. (2006). Dispersal of fig pollinators in Asian tropical rain forests. Journal of Tropical Ecology, 22, 631–639.

[ece39311-bib-0036] Herre, E. A. (1985). Sex ratio adjustment in fig wasps. Science, 228, 896–898. 17815055 10.1126/science.228.4701.896

[ece39311-bib-0037] Herre, E. A. (1989). Coevolution of reproductive characteristics in 12 species of New World figs and their pollinator wasps. Experientia, 45, 637–647.

[ece39311-bib-0038] Herre, E. A. , Jandér, K. C. , & Machado, C. A. (2008). Evolutionary ecology of figs and their associates: Recent progress and outstanding puzzles. Annual Review of Ecology, Evolution, and Systematics, 39, 439–458.

[ece39311-bib-0039] Hubbell, S. , Comita, L. , Lao, S. , & Condit, R. (2014). Barro Colorado fifty hectare plot census of canopy density 1983‐2012. Center of Tropical Forest Sciences Databases.

[ece39311-bib-0040] IPBES . (2016). The assessment report of the intergovernmental science‐policy platform on biodiversity and ecosystem services on pollinators, pollination and food production. In S. G. Potts , V. L. Imperatriz‐Fonseca , & H. T. Ngo (Eds.), Secretariat of the Intergovernmental Science‐Policy Platform on Biodiversity and Ecosystem Services (p. 36). IPBES.

[ece39311-bib-0041] IPCC . (2021). Summary for policymakers. In V. Masson‐Delmotte , P. Zhai , A. Pirani , S. L. Connors , C. Péan , S. Berger , N. Caud , Y. Chen , L. Goldfarb , M. I. Gomis , M. Huang , K. Leitzell , E. Lonnoy , J. B. R. Matthews , T. K. Maycock , T. Waterfield , O. Yelekçi , R. Yu , & B. Zhou (Eds.), Climate change 2021: The physical science basis. Contribution of working group I to the sixth assessment report of the Intergovernmental Panel on Climate Change (pp. 3–32). Cambridge University Press.

[ece39311-bib-0042] Jandér, K. C. , Dafoe, A. , & Herre, E. A. (2016). Fitness reduction for uncooperative fig wasps through reduced offspring size: A third component of host sanctions. Ecology, 97, 2491–2500. 27859079 10.1002/ecy.1471

[ece39311-bib-0043] Jandér, K. C. , Herre, E. A. , & Simms, E. L. (2012). Precision of host sanctions in the fig tree‐fig wasp mutualism: Consequences for uncooperative symbionts. Ecology Letters, 15, 1362–1369. 22925044 10.1111/j.1461-0248.2012.01857.x

[ece39311-bib-0044] Jandér, K. C. , & Steidinger, B. S. (2017). Why mutualist partners vary in quality: Mutation–selection balance and incentives to cheat in the fig tree–fig wasp mutualism. Ecology Letters, 20, 922–932. 28612473 10.1111/ele.12792

[ece39311-bib-0045] Jevanandam, N. , Goh, A. G. R. , & Corlett, R. T. (2013). Climate warming and the potential extinction of fig wasps, the obligate pollinators of figs. Biology Letters, 9, 20130041. 23515979 10.1098/rsbl.2013.0041 PMC3645034

[ece39311-bib-0046] Käfer, H. , Kovac, H. , & Stabentheiner, A. (2012). Upper thermal limits of honeybee (*Apis mellifera*) and yellowjacket (*Vespula vulgaris*) foragers. Mitteilungen der Deutschen Gesellschaft für Allgemeine und Angewandte Entomologie, 18, 267–270.

[ece39311-bib-0047] Kassambara, A. , Kosinski, M. , Biecek, P. , & Scheipl, F. (2019). Survminer: Survival analysis and visualization. *R package version 0.4. 7* .

[ece39311-bib-0048] Kearns, C. A. , Inouye, D. W. , & Waser, N. M. (1998). Endangered mutualisms: The conservation of plant‐pollinator interactions. Annual Review of Ecology and Systematics, 29, 83–112.

[ece39311-bib-0049] Kiers, T. E. , Palmer, T. M. , Ives, A. R. , Bruno, J. F. , & Bronstein, J. L. (2010). Mutualisms in a changing world: An evolutionary perspective. Ecology Letters, 13, 1459–1474. 20955506 10.1111/j.1461-0248.2010.01538.x

[ece39311-bib-0050] Kirtman, B. , Power, S. B. , Adedoyin, A. J. , Boer, G. J. , Bojariu, R. , Camilloni, I. , Doblas‐Reyes, F. , Fiore, A. M. , Kimoto, M. , Meehl, G. , Prather, M. , Sarr, A. , Schär, C. , Sutton, R. , van Oldenborgh, G. J. , Vecchi, G. , & Wang, H. J. (2013). Near‐term climate change: Projections and predictability. In T. F. Stocker , D. Qin , G.‐K. Plattner , M. Tignor , S. K. Allen , J. Boschung , A. Nauels , Y. Xia , V. Y. Xia , V. Bex , & P. M. Midgley (Eds.), Climate change 2013 the physical science basis: Working group I contribution to the fifth assessment report of the Intergovernmental Panel on Climate Change. Cambridge University Press.

[ece39311-bib-0051] Kordas, R. L. , Harley, C. D. G. , & O'Connor, M. I. (2011). Community ecology in a warming world: The influence of temperature on interspecific interactions in marine systems. Journal of Experimental Marine Biology and Ecology, 400, 218–226.

[ece39311-bib-0052] Kumagai, T. , Kuraji, K. , Noguchi, H. , Tanaka, Y. , Tanaka, K. , & Suzuki, M. (2001). Vertical profiles of environmental factors within tropical rainforest, Lambir Hills National Park, Sarawak, Malaysia. Journal of Forest Research, 6, 257–264.

[ece39311-bib-0053] Machado, C. A. , Robbins, N. , Gilbert, M. T. P. , & Herre, E. A. (2005). Critical review of host specificity and its coevolutionary implications in the fig/fig‐wasp mutualism. Proceedings of the National Academy of Sciences of the United States of America, 102, 6558–6565. 15851680 10.1073/pnas.0501840102 PMC1131861

[ece39311-bib-0054] Maebe, K. , De Baets, A. , Vandamme, P. , Vereecken, N. J. , Michez, D. , & Smagghe, G. (2021). Impact of intraspecific variation on measurements of thermal tolerance in bumble bees. Journal of Thermal Biology, 99, 103002. 34420633 10.1016/j.jtherbio.2021.103002

[ece39311-bib-0055] Maia‐Silva, C. , da Silva Pereira, J. , Freitas, B. M. , & Hrncir, M. (2021). Don't stay out too long! Thermal tolerance of the stingless bees *Melipona subnitida* decreases with increasing exposure time to elevated temperatures. Apidologie, 52, 218–229.

[ece39311-bib-0056] Marussich, W. A. , & Machado, C. A. (2007). Host‐specificity and coevolution among pollinating and nonpollinating New World fig wasps. Molecular Ecology, 16, 1925–1946. 17444902 10.1111/j.1365-294X.2007.03278.x

[ece39311-bib-0057] Mawdsley, N. A. , Compton, S. G. , & Whittaker, R. J. (1998). Population persistence, pollination mutualisms, and figs in fragmented tropical landscapes. Conservation Biology, 12, 1416–1420.

[ece39311-bib-0058] Mech, A. M. , Tobin, P. C. , Teskey, R. O. , Rhea, J. R. , & Gandhi, K. J. K. (2018). Increases in summer temperatures decrease the survival of an invasive forest insect. Biological Invasions, 20, 365–374.

[ece39311-bib-0059] Memmott, J. , Craze, P. G. , Waser, N. M. , & Price, M. V. (2007). Global warming and the disruption of plant‐pollinator interactions. Ecology Letters, 10, 710–717. 17594426 10.1111/j.1461-0248.2007.01061.x

[ece39311-bib-0060] Molbo, D. , Machado, C. A. , Herre, E. A. , & Keller, L. (2004). Inbreeding and population structure in two pairs of cryptic fig wasp species. Molecular Ecology, 13, 1613–1623. 15140104 10.1111/j.1365-294X.2004.02158.x

[ece39311-bib-0061] Nasir, M. , Ata‐Ul‐Mohsan, M. , Ahmad, S. , Saeed, M. , Aziz, A. , Imran, M. , & Sheikh, U. A. A. (2019). Effect of different temperatures on colony characteristics of *Bombus terrestris* (Hymenoptera: Apidae). Pakistan Journal of Zoology, 51, 1315–1322.

[ece39311-bib-0062] Nason, J. D. , Herre, E. A. , & Hamrick, J. L. (1996). Paternity analysis of breeding structure of strangler fig populations: Evidence for substantial long‐distance wasp dispersal. Journal of Biogeography, 23, 501–512.

[ece39311-bib-0063] Nason, J. D. , Herre, E. A. , & Hamrick, J. L. (1998). The breeding structure of a tropical keystone plant resource. Nature, 391, 685–687.

[ece39311-bib-0064] Ollerton, J. , Winfree, R. , & Tarrant, S. (2011). How many flowering plants are pollinated by animals? Oikos, 120, 321–326.

[ece39311-bib-0065] Oyen, K. J. , & Dillon, M. E. (2018). Critical thermal limits of bumblebees (*Bombus impatiens*) are marked by stereotypical behaviors and are unchanged by acclimation, age or feeding status. Journal of Experimental Biology, 221, jeb165589. 29530975 10.1242/jeb.165589

[ece39311-bib-0066] Patiño, S. , Herre, E. A. , & Tyree, M. T. (1994). Physiological determinants of *Ficus* fruit temperature and implications for survival of pollinator wasp species: Comparative physiology through an energy budget approach. Oecologia, 100, 13–20. 28307022 10.1007/BF00317125

[ece39311-bib-0067] Paton, S. (2020). Yearly reports_Barro Colorado Island. The Smithsonian Institution. Dataset. 10.25573/data.11799111.v1

[ece39311-bib-0068] Potts, S. G. , Biesmeijer, J. C. , Kremen, C. , Neumann, P. , Schweiger, O. , & Kunin, W. E. (2010). Global pollinator declines: Trends, impacts and drivers. Trends in Ecology & Evolution, 25, 345–353. 20188434 10.1016/j.tree.2010.01.007

[ece39311-bib-0069] Robertson, A. W. , Kelly, D. , Ladley, J. J. , & Sparrow, A. D. (1999). Effects of pollinator loss on endemic New Zealand mistletoes (Loranthaceae). Conservation Biology, 13, 499–508.

[ece39311-bib-0070] Sánchez‐Bayo, F. , & Wyckhuys, K. A. G. (2019). Worldwide decline of the entomofauna: A review of its drivers. Biological Conservation, 232, 8–27.

[ece39311-bib-0071] Sánchez‐Echeverría, K. , Castellanos, I. , Mendoza‐Cuenca, L. , Zuria, I. , & Sánchez‐Rojas, G. (2019). Reduced thermal variability in cities and its impact on honey bee thermal tolerance. PeerJ, 2019, 1–17. 10.7717/peerj.7060 PMC6557256 31211017

[ece39311-bib-0072] Santinelo Pereira, R. A. , De Pádua Teixeira, S. , & Kjellberg, F. (2007). An inquiline fig wasp using seeds as a resource for small male production: A potential first step for the evolution of new feeding habits? Biological Journal of the Linnean Society, 92, 9–17.

[ece39311-bib-0073] Satler, J. D. , Allen Herre, E. , Heath, T. A. , Machado, C. A. , Gómez Zúñiga, A. , & Nason, J. D. (2020). Genome‐wide sequence data show no evidence of admixture and introgression among pollinator wasps associated with a community of Panamanian strangler figs. bioRxiv. 10.1101/2020.12.09.418376 PMC9545327 35090071

[ece39311-bib-0074] Shanahan, M. , Samson, S. O. , Compton, S. G. , & Corlett, R. (2001). Fig‐eating by vertebrate frugivores: A global review. Biological Reviews of the Cambridge Philosophical Society, 76, 529–572. 11762492 10.1017/s1464793101005760

[ece39311-bib-0075] Sunday, J. M. , Bates, A. E. , Kearney, M. R. , Colwell, R. K. , Dulvy, N. K. , Longino, J. T. , & Huey, R. B. (2014). Thermal‐safety margins and the necessity of thermoregulatory behavior across latitude and elevation. Proceedings of the National Academy of Sciences of the United States of America, 111, 5610–5615. 24616528 10.1073/pnas.1316145111 PMC3992687

[ece39311-bib-0076] Suttle, K. B. , Thomsen, M. A. , & Power, M. E. (2007). Species interactions reverse grassland responses to changing climate. Science, 315, 640–642. 17272720 10.1126/science.1136401

[ece39311-bib-0077] Sutton, T. L. , DeGabriel, J. L. , Riegler, M. , & Cook, J. M. (2018). A temperate pollinator with high thermal tolerance is still susceptible to heat events predicted under future climate change. Ecological Entomology, 43, 506–512.

[ece39311-bib-0078] Therneau, T. M. , Lumley, T. , Atkinson, E. , & Crowson, C. (2020). Survival: Survival Analysis. *R package version 3.1‐11* .

[ece39311-bib-0079] Therneau, T. M. (2018). Coxme: Mixed effects cox models. *R Package version 2.2‐10* .

[ece39311-bib-0080] Todzia, C. (1986). Growth habits, host tree species, and density of hemiepiphytes on Barro Colorado Island, Panama. Biotropica, 18, 22.

[ece39311-bib-0081] Van Goor, J. , Piatscheck, F. , Houston, D. D. , & Nason, J. D. (2018). Figs, pollinators, and parasites: A longitudinal study of the effects of nematode infection on fig wasp fitness. Acta Oecologica, 90, 140–150.

[ece39311-bib-0082] Van Goor, J. , Piatscheck, F. , Houston, D. D. , & Nason, J. D. (2021). Differential effects of nematode infection on pollinating and non‐pollinating fig wasps: Can shared antagonism provide net benefits to a mutualism? Journal of Animal Ecology, 90, 1764–1775. 33934356 10.1111/1365-2656.13495

[ece39311-bib-0083] Van Swaay, C. , Van Strien, A. , Aghababyan, K. , Astrom, S. , Botham, M. , Brereton, T. , Chambers, P. , Collins, S. , Domenech Ferre, M. , Escobes, R. , Feldmann, R. , Fernandez‐Garcia, J. M. , Fontaine, B. , Goloshchapova, S. , Gracianteparaluceta, A. , Harpke, A. , Heliola, J. , Khanamirian, G. , Julliard, R. , … Warren, M. (2015). The European butterfly indicator for grassland species: 1990‐2013. VS2015.009. Wageningen.

[ece39311-bib-0084] Vanbergen, A. J. , & Garratt, M. P. (2013). Threats to an ecosystem service: Pressures on pollinators. Frontiers in Ecology and the Environment, 11, 251–259.

[ece39311-bib-0085] Vidal, M. C. , Anneberg, T. J. , Curé, A. E. , Althoff, D. M. , & Segraves, K. A. (2021). The variable effects of global change on insect mutualisms. Current Opinion in Insect Science, 47, 46–52. 33771734 10.1016/j.cois.2021.03.002

[ece39311-bib-0086] Wagner, D. L. , Grames, E. M. , Forister, M. L. , Berenbaum, M. R. , & Stopak, D. (2021). Insect decline in the Anthropocene: Death by a thousand cuts. Proceedings of the National Academy of Sciences of the United States of America, 118, 1–10. 10.1073/pnas.2023989118 PMC7812858 33431573

[ece39311-bib-0087] Ware, A. B. , & Compton, S. G. (1994). Dispersal of adult female fig wasps: 2. Movements between trees. Entomologia Experimentalis et Applicata, 73, 231–238.

[ece39311-bib-0088] West, S. A. , & Herre, E. A. (1994). The ecology of the New World fig‐parasitizing wasps *Idarnes* and implications for the evolution of the fig‐pollinator mutualism. Proceedings of the Royal Society B: Biological Sciences, 258, 67–72.

[ece39311-bib-0089] West, S. A. , Herre, E. A. , Windsor, D. M. , & Green, P. R. S. (1996). The ecology and evolution of the New World non‐pollinating fig wasp communities. Journal of Biogeography, 23, 447–458.

[ece39311-bib-0090] Wiebes, J. T. (1979). Co‐evolution of figs and their insect pollinators. Annual Review of Ecology and Systematics, 10, 1–12.

[ece39311-bib-0091] Wiebes, J. T. (1995). Agaonidae (Hymenoptera Chalcidoidea) and *Ficus* (Moraceae): Fig wasps and their figs, xv (Meso‐American Pegoscapus). Proceedings of the Koninklijke Nederlandse Akademie van Wetenschappen, 98, 167–183.

[ece39311-bib-0092] Wynants, E. , Lenaerts, N. , Wäckers, F. , & van Oystaeyen, A. (2021). Thermoregulation dynamics in commercially reared colonies of the bumblebee *Bombus terrestris* . Physiological Entomology, 46, 110–118.

[ece39311-bib-0093] Zattara, E. E. , & Aizen, M. A. (2021). Worldwide occurrence records suggest a global decline in bee species richness. One Earth, 4, 114–123.

